# Laying the foundation in food allergy education: CSACI food allergy educator program

**DOI:** 10.1186/s13223-026-01019-z

**Published:** 2026-04-10

**Authors:** Jennifer L. P. Protudjer, Douglas P. Mack, Lori Connors, Jasmin Lidington, Harold Kim

**Affiliations:** 1 Children’s Hospital Research Institute of Manitoba, 501G-715 McDermot Avenue, Winnipeg, MB R3E 3P4 Canada; 2 Department of Pediatrics and Child Health, The University of Manitoba, Winnipeg, Canada; 3 Department of Food and Human Nutritional Sciences, The University of Manitoba, Winnipeg, Canada; 4 Institute for Environmental Medicine, Karolinska Institutet, Stockholm, Sweden; 5 McMaster University, Hamilton, ON Canada; 6 Dalhousie University, Halifax, NS Canada; 7 Canadian Society of Allergy and Clinical Immunology, Ottawa, ON Canada; 8 University of Western Ontario, Kitchener, ON Canada

**Keywords:** Food allergy, Medical education

## Abstract

In Fall 2023, the Canadian Society of Allergy and Clinical Immunology (CSACI) introduced an evidence-based, participant-informed food allergy educator program for physicians and allied health professionals working in allergy. Herein, we aimed to provide an overview and evaluation of the first four cohorts of the foundational course. The 8-weeks foundational course in food allergy, the content of which was delivered by world leaders in food allergy, included topics ranging from food allergy epidemiology, diagnosis and management to the psychosocial burden of food allergy. Learners completed a pre-test and a post-test, one week prior to the course, and one week following the course, respectively. We operationalized the passing grade for the post-test as a minimum of 75%. Data were analysed descriptively with comparisons made using a paired t-test (Stata Version 19.0, College Station, TX). To date, four cohorts, including 34 learners, have completed the foundational course. The pre-test and post-test mean scores were 72.5% (range 30.0–96.0%) and 84.5% (range 65.0–97.5%), respectively, which corresponds to a significant improvement in food allergy knowledge from the pre-test (*p* < 0.0001). in the first four cohorts of the CSACI Food Allergy Educator Program Foundational Course, food allergy knowledge significantly improved among learners. The foundational course will continue to be offered at least once yearly. An advanced practice course is also being developed.

To the Editor:

The prevalence of food allergy continues to rise globally [1]. Despite this, there is a notable lack of food allergy education in the curricula of many health professions. As a result, health professionals may encounter patients with food allergies yet may not receive the evidence-based food allergy education during academic training necessary to provide high-quality care. Indeed, allied healthcare professionals have reported being afraid to work with this patient population [2], while non-allergist medical doctors have indicated a desire to embed such training in their mandatory learning [3]. As part of the 76th annual meeting of the Canadian Society of Allergy and Clinical Immunology (CSACI), we hosted a virtual workshop to seek guidance on the need for a food allergy educator program in Canada [4]. In response to the need for continued professional development training in allergy, the CSACI introduced a food allergy educator program (CFAE) for professionals working in food allergy in Fall 2023. In this submission, we present an update on the first three iterations of the foundational course.

In Fall 2023, we launched an 8-week foundational course in food allergy, which has since been delivered in Spring 2024, Fall 2024 and Spring 2025. The content was informed based on feedback received during the above-described virtual workshop [4], coupled with research to provide supports that address the psychosocial [5, 6] and financial burdens of food allergy [7], as well as primary prevention [8] and emerging food allergy therapies, such as oral immunotherapy [9]. The CFAE leadership identified prospective faculty based on global leadership in food allergy and the alignment of their areas of expertise and our program. Similarly, the CFAE leadership aimed to construct our faculty based on the principles of equity, diversity and inclusion. Each session’s 8–10 faculty represented approximate gender parity and diverse ethnicities.

To date, the course has exclusively been advertised through the CSACI. Learners were required to be professionals working in food allergy. Prospective learners applied to the program with basic demographic details, such as province, years of food allergy practice, and the highest degree. Once accepted, learners completed a pre-test approximately one week before course commencement.

Over an 8-week course, learners met once weekly for approximately an hour, during which faculty delivered a synchronous lecture with defined intended learning outcomes (ILOs). ILOs were constructed by CFAE leadership and guided by Bloom’s Taxonomy, a widely used framework to assist educators with the structuring of curricula and to address different learning styles [10]. Faculty welcomed questions of clarification or further detail during and/or after their synchronous lectures.

In addition to 8 once-weekly synchronous lectures, learners had access to pre-readings for each session, which were located within the password-protected area of the course website (https://foodallergyeducation.ca/).

One week following the completion of the course, learners completed a closed book post-test, for which the passing grade was 75%. During the post-test period, learners did not have access to the pre-readings. Of note, all faculty contributed single best choice multiple choice questions to both the pre-test and the post-test. Test results were communicated to each learner by a member of CFAE leadership. Learners who passed the post-test were awarded a certificate of successful completion. This certificate includes a legal disclaimer that that successful completion of the course does not imply any certification or qualification in the field of allergology or immunology, or in any other medical specialty.

To analyse improvements from pre-test to post-test food allergy knowledge, we performed a series of statistical analyses. Data were analysed descriptively with comparisons made using paired t-test and analysis of variance (ANOVA; Stata Version 19.0, College Station, TX). As an educational course, the University of Manitoba Research Ethics Board deemed research ethics board approval unnecessary subsequent to the review of the course proposal and overview (HS25785 (H2022:389)).

A total of 34 learners were involved in the first four cohorts of the CFAE Foundational Course. Of these, slightly fewer than half (14/34; 43.8%) were nurses. With consideration to all learners combined, the mean pre-test score (converted to percentages) was 72.5% (range 30.0–96.0%) and the mean post-test score (converted to percentages) was 84.5% (range 65.0–97.0%), corresponding to a significant overall improvement in foundational food allergy knowledge (*p* < 0.0001). The 40 post-test questions addressed four different domains: dietary management, cross-contamination, and cross-reactivity (*n* = 8 questions); concerns in infancy (*n* = 4 questions); diagnostics and oral immunotherapy (*n* = 14 questions); and, day-to-day management (*n* = 14 questions). There was no statistically significant differences in learner success between the domains. Within the domain, day-to-day management, only one-quarter were able to appropriately identify the pattern of food allergy prevalence (8/34; 23.5%).

With consideration given to learner-specific professions, pre-test scores did not significantly differ. Similar to the increase seen for all learners combined, the improvement in scores persisted when stratified by type of professional group. Significant improvements in foundational knowledge were noted for nurses (*p* = 0.05) and medical doctors (*p* = 0.03); and trended towards a significant improvement for registered dietitians (*p* = 0.12). Post-test scores (converted to percentages) ranged from 82.25 ± 2.94 for registered dietitians, to 91.25 ± 1.25, for medical doctors. However, between-profession post-test score differences were not statistically significant.

In summary, the first four iterations of our evidence-based foundational course on food allergy education for professionals working in the food allergy space significantly improved learners’ knowledge from the start to the end of the program. Looking to the future, we will further expand advertising to health professionals across Canada in effort to train as many as possible in food allergy. We will also launch the advanced practice course, intended for those who have successfully completed the foundational course and wish to gain deeper knowledge of food allergy. Such steps are essential to support an increasing population of individuals and their families managing food allergy [1], and to provide additional trained expertise for allied health professionals supporting the less than 300 allergists currently practicing in Canada [11].

## Data Availability

The datasets used and/or analysed during the current study are available from the corresponding author on reasonable request.

## Abbreviations

ANOVA: Analyses of variance
CFAE: CSACI Food Allergy Educator
CSACI: Canadian Society of Allergy and Clinical Immunology
ILOs: Intended learning outcome(s)
